# Differential effects of the translocator protein 18 kDa (TSPO) ligand etifoxine and the benzodiazepine alprazolam on startle response to predictable threat in a NPU-threat task after acute and short-term treatment

**DOI:** 10.1007/s00213-022-06111-x

**Published:** 2022-03-12

**Authors:** Lisa-Marie Brunner, Franziska Maurer, Kevin Weber, Johannes Weigl, Vladimir M. Milenkovic, Rainer Rupprecht, Caroline Nothdurfter, Andreas Mühlberger

**Affiliations:** 1 Department of Medicine, Psychiatry and Psychotherapy, University Regensburg, 93053 Regensburg, Germany; 2 Department of Psychology, Clinical Psychology and Psychotherapy, University Regensburg, Regensburg, Germany

**Keywords:** GABA_A_ receptor, Translocator protein 18 kDa (TSPO), NPU-threat task, Predictable threat, Unpredictable threat, Etifoxine, Alprazolam

## Abstract

**Rationale:**

Benzodiazepines have been extensively investigated in experimental settings especially after single administration, which mostly revealed effects on unpredictable threat (U-threat) rather than predictable threat (P-threat). Given the need for pharmacological alternatives with a preferable side-effect profile and to better represent clinical conditions, research should cover also other anxiolytics and longer application times.

**Objectives:**

The present study compared the acute and short-term effects of the translocator protein 18 kDa (TSPO) ligand etifoxine and the benzodiazepine alprazolam on P-threat and U-threat while controlling for sedation.

**Methods:**

Sixty healthy male volunteers, aged between 18 and 55 years, were randomly assigned to receive a daily dose of either 150 mg etifoxine, 1.5 mg alprazolam, or placebo for 5 days. On days 1 and 5 of intake, they performed a NPU-threat task including neutral (N), predictable (P), and unpredictable (U) conditions, while startle responsivity and self-reports were studied. Sedative effects were assessed using a continuous performance test.

**Results:**

Neither alprazolam nor etifoxine affected startle responsivity to U-threat on any of the testing days. While etifoxine reduced the startle response to P-threat on day 1 of treatment for transformed data, a contrary effect of alprazolam was found for raw values. No effects on self-reports and no evidence of sedation could be observed for either drug.

**Conclusions:**

None of the anxiolytic substances had an impact on startle potentiation to U-threat even after several days of intake. The effects of the anxiolytics on startle responsivity to P-threat as well as implications for future studies are discussed.

**Supplementary Information:**

The online version contains supplementary material available at 10.1007/s00213-022-06111-x.

## Introduction

Benzodiazepines rank among the most frequently prescribed substances for the treatment of anxiety disorders (Donoghue and Lader, 2010). While being effective and fast acting, they bear the risk of side effects including sedation, risk of addiction potential, and withdrawal symptoms, especially after long-term application (Lader, 2011). Therefore, they are not considered as first-choice treatments like antidepressants or psychotherapy (Bandelow et al. 2015). With regard to the potential for the pharmacological treatment of anxiety disorders, novel alternatives should aim for combining the efficacy and fast onset of existing compounds with lacking the most critical side effects.

Interesting candidates in this context are ligands of the translocator protein 18 kDa (TSPO) (Papadopoulos et al. 2006; Nothdurfter et al. 2012; Rupprecht et al. 2009, 2010). A role in anxiolysis has been derived from its location at the outer membrane of mitochondria, where it is involved in the local cholesterol transport and subsequent synthesis of anxiolytic neurosteroids (Krueger and Papadopoulos, 1990). Besides expression in central (micro-)glia and reactive astrocytes, TSPO is detectable in peripheral tissue qualifying it as a biomarker for pathological conditions of the organism applicable in clinical settings (Cosenza-Nashat et al. 2009). Indeed, peripheral expression of TSPO differentiated between depressive patients with comorbid separation anxiety and healthy controls (Chelli et al. 2008; Abelli et al. 2010) and also subjects with high trait anxiety within a healthy sample (Nakamura et al. 2002). In line with that, genetically determined disruption of TSPO function has been shown to predispose separation anxiety (Costa et al. 2009) or bipolar disorder (Colasanti et al. 2013).

Preclinical research on ligands binding to TSPO like the benzoxazine derivate etifoxine reported increased neurosteroidogenesis in cells (Wolf et al. 2015) as well as reduction of stress-related reactions in animals (Verleye and Gillardin 2004). Similarly, in patients suffering from adjustment disorder with anxiety, etifoxine reduced clinical symptoms to a comparable amount as benzodiazepines, however, lacking their sedative side effects (Nguyen et al. 2006; Stein 2015; Micallef et al. 2001). A further interesting point on etifoxine is its twofold action mechanism. Besides its action on TSPO, it directly binds to the GABA_A_ receptor, thereby modulating transmission of the inhibitory neurotransmitter GABA (Hamon et al. 2003). Its association to the β-subunit of the receptor — in contrast to benzodiazepines that bind to the α/γ-subunits — might thereby explain its preferable profile of side effects (Sieghart & Sperk, 2002; Möhler, 2012). However, so far, human studies on etifoxine investigated specific pathological conditions of anxiety only, and measurement of effects solely comprised reports of patients or health professionals. Thus, to gain deeper insights into its mechanisms of action, and to gain more insight about the comparability to established treatment, placebo-controlled experimental studies are needed.

One state-of-the-art paradigm for the investigation of the effects of anxiolytic compounds in animal and human research is the NPU-threat task (Schmitz & Grillon, 2012). By varying the predictability of the aversive stimuli using specific cues, predictable (P) threat responding (related to phasic fear) and unpredictable (U) threat responding (related to sustained anxiety) can be evoked and contrasted to a neutral (N) condition (Davis et al. 2010; Schmitz & Grillon, 2012). Meanwhile, the modulation of startle reactivity in response to an abrupt, intense noise is measured (Grillon 2008). Manifold research exists that measured startle response to identify anxiolytic effects of benzodiazepines with either showing an attenuation of responses to U-threat but not P-threat (Grillon et al. 2006; Baas et al. 2002) or unspecific effects on overall baseline startle (Baas et al. 2009; Acheson et al. 2012). Criticism that attenuating effects of benzodiazepines might rather arise due to sedation than to anxiolysis has been rebutted by work including a sedative non-anxiolytic control, which only affected baseline startle response without any specific anxiety-related effects (Grillon et al. 2006).

Due to its capacity to measure parameters related to fear and anxiety within one paradigm while controlling overall sedation effects, the NPU threat task is a perfect paradigm to experimentally compare the anxiolytic effects of the TSPO ligand etifoxine to the benzodiazepine alprazolam. In contrast to previous research on benzodiazepines, we did not only assess acute effects after intake of a single dose but also after 5 days of medication resembling a subchronic use, based on an analysis that revealed startle reactivity to be a suitable tool for repeated measurement (Klumpers et al. 2010). Based on the findings of similar anxiolytic efficacy in patient samples and the hypothesized overlap of responses to U-threat and generalized anxiety disorder, a similar profile as for benzodiazepines could be expected for etifoxine. To test the assumption of larger sedative effects of alprazolam compared to etifoxine and placebo, we administered a sustained attention task on both testing days directly before the startle paradigm. Within the etifoxine group, we further checked for the possible impact of the TSPO gene polymorphism rs6971, which leads to structural changes of the protein structure (Owen et al. 2012), affects the affinity with which ligands bind to the protein, and might thereby influence their therapeutic effects (Owen et al. 2011).

## Methods and procedure

### Study design

The present work was part of a randomized controlled double-blind clinical trial, which was conducted in cooperation between the Department of Psychiatry and Psychotherapy and the Department of Psychology (Clinical Psychology and Psychotherapy) at the University of Regensburg from July 2018 to November 2019. The trial complied with the Declaration of Helsinki, the Guidelines for Good Clinical Practice of the International Conference on Harmonization, and the legal requirements of the German Medicine Law for Clinical Trials. The ethics committee of the University of Regensburg and the Federal Institute for Drugs and Medical Devices (BfArM) approved the study plan. The clinical trial was registered at the Clinical Trials Register (EudraCT number: 2016–004,254-15), the German Register of Clinical Studies (DRKS number: DRKS00023318), and the regional authorities (government of Upper Franconia). All participants gave informed consent and were paid 500 € in case of study completion.

### Participants

We included 60 healthy male participants aged between 18 and 55 years into the trial. The gender criterion was due to hormonal measures in relation to a stress test, which we reported elsewhere (Bahr et al. 2021). Mental health was assessed using the Mini International Neuropsychiatric Interview (MINI) (ver. 5.0.0; Sheehan et al. 1998) with any current DSM diagnosis as well as alcohol or drug dependence during lifetime prohibiting participation in the study. This was followed by an examination conducted by a physician according to standards of the clinical routine as well as the assessment of vital and blood parameters with a special focus on liver and kidney function. Eligibility criteria on physical health were oriented on a consensus report of BfArM (Breithaupt-Groegler et al. 2017). Further inclusion criteria were the ability to conceive the nature and meaning of the clinical trial and the willingness to forgo the consumption of alcohol and other drugs, driving a car, and the operation of heavy machines during participation in the study. A urine sample was taken during the screening to rule out current drug consumption. Exclusion criteria were contraindications or hypersensitivity against the study medication, concurrent participation in another pharmacological trial, and intake of psychotropic medication within the last 6 months.

### Material and measures

#### Drug treatment

Participants were randomly assigned to receive either a daily dose of 1.5 mg alprazolam (0.5–0.5–0.5 mg), 150 mg etifoxine (50–50–50 mg), or placebo (only filler mixture, no active substances). While alprazolam reaches plasma peak concentrations within 1 to 2 h, maximal concentration in blood of etifoxine is reached after 2 to 3 h. Elimination half-lives of alprazolam and etifoxine are 9 to16 h and 2 to 6 h, respectively. The respective doses of the medication were based on the recommended amount for the use in patients as well as on previous research reporting similar efficacy of the two compounds (Stein, 2015). All treatments were divided into three doses (8:00 AM, 12:00 PM, 6:00 PM) and given for 5 days in total to reach subchronic levels but not to risk addiction or withdrawal symptoms at the end of the treatment. Medication was provided as capsules that looked identical for all three groups for oral intake that were prepared by the pharmacy of the University of Erlangen.

#### NPU-threat task

P-threat and U-threat responding was assessed using the NPU-threat task (for a detailed description of the experimental protocol, see Schmitz and Grillon, 2012) (Presentation, version 19.0, Neurobehavioral Systems Inc., Albany, California, USA). The task consisted of three conditions, which were indicated by geometrical cues with the following scheme: neutral (N): green circle = no stimulation; predictable (P): red square = stimulation only during presence of the cue; and unpredictable (U): blue triangle = stimulation at any time independent of the cue. The experiment consisted of two 15-min blocks, which differed according to the presentation scheme: U N P N P N U or P N U N U N P. Participants were assigned randomly to start with either one or the other order at the two testing days. During each condition (120-s duration), the respective geometrical cues were presented three times for periods of 8 s alternating with phases, during which only the text describing the condition was presented (ranging from 20 to 37 s). Within the task, electric stimulation (100 ms duration) applied by a constant-current stimulator (Digitimer DS7A; Digitimer, Hertfordshire, UK) at the upper side of the right forearm served as aversive stimulus. The strength was adjusted individually for every participant using the QUEST procedure (Onat & Büchel, 2015) slightly modified with choosing a value 1.3 times the rated threshold between unpleasantness and pain. In total, each participant received 12 electric shocks (6 in P, 6 in U) during the task. Independently of the electric stimulation startle, reactivity was evoked by bursts of white noise at 103 dB (40-ms duration) via headphones (Sennheiser HD 569, Sennheiser electronic GmbH & Co. KG, Wedemark-Wennebostel, Germany). The time interval between a startle probe and a preceding startle or electric shock was always greater than 15 s to ensure that the startle response was not significantly potentiated by an immediately preceding stimulus.

#### Self-reports

The State-Trait Anxiety Inventory (STAI trait) assesses anxiety (20 items, scale from 1 — *not at all* to 4 — *very much so*) (Laux et al. 1981). The Anxiety Sensitivity Index-3 (ASI-3) measures the trait variable anxiety sensitivity (18 items, scale from 0 = *very little* to 4 = *very much* (Kemper et al. 2009). The Intolerance of Uncertainty Scale (IUS-18) quantifies the degree to which ambiguous or uncertain situations are experienced as unpleasant (18 items, scale from 1 = *not at all characteristic of me* to 5 = *entirely characteristic of me*) (Gerlach et al. 2008).

We further administered a questionnaire on the NPU-threat task (Schmitz and Grillon 2012), in which participants stated the level of experienced anxiety (from 1 — *not anxious* to 10 — *very anxious*) during the three conditions separately for the time windows when the respective cue was present or absent.

#### Startle response

Startle responses were digitally amplified (V-Amp, Brain Products GmbH, Gilching, Germany) and recorded at a sampling rate of 1000 Hz (Brain Vision Recorder, Brain Products GmbH, Gilching, Germany). We used two surface electromyographic electrodes at the left orbicularis oculi muscle as well as ground and reference electrodes placed at the mastoids.

#### Continuous Performance Test (CPT-AX)

To check for sedating effects, we applied the Continuous Performance Test (CPT, AX-version; Servan-Schreiber et al. 1996) (Presentation, version 19.0, Neurobehavioral Systems Inc., Albany, California, USA). In this task, subjects were prompted to respond differently to target letters (X following an A; AX) and nontarget trials with either any other letter following the cue (e.g., AY), any other letter preceding the probe (BX), or a combination of two letters including neither the probe nor the cue (BY). The task duration was about 25 min divided into four blocks of equal length. The different sequences were shown in a pseudo-randomized order with the following probabilities: 70% AX trials (*n* = 126 per block), 10% AY trials (*n* = 18 per block), 10% BX trials (*n* = 18 per block), and 10% BY trials (*n* = 18 per block) with a stimulus duration of 250 ms followed by an interstimulus interval of 800 ms.

### Procedure

After signing informed consent, eligibility of study prospects was assessed within a screening, which had to be scheduled no longer than 7 days before the planned start of participation. Besides completion of the trait questionnaires, blood samples were taken for the determination of the TSPO gene polymorphism rs6971. Furthermore, participants performed the CPT-AX for the first time, which was repeated on days 1 and 5 of treatment at about 1/2 h after intake of the first medication dose in the morning taken at 8:00 AM. Each time, participants first received information on the task and had to pass a practice phase. At the two treatment days, participants had a break of 30 min after the CPT-AX before they returned for the NPU-threat test at around 10:30 AM. Again, they were provided information on the experimental conditions followed by attachment of the electrodes for the physiological recordings as well as for the electrical stimulation after disinfection and peeling the respective skin areas. To avoid an influence of excessive startle reactivity at the beginning, we first applied a habituation phase with four presentations of the white noise tone. Before starting the actual experiment, participants were told to move as little as possible from then on to avoid artifacts of the physiological measurements. After the first block as well as at the end (around 11:30 AM), the questionnaire on experienced anxiety during the NPU-threat task had to be filled out.

### Data preprocessing

*Startle response*: Startle data were preprocessed with the BrainVision Analyzer (Version 2.1, Brain Products GmbH, Germany) applying the following filters: low cutoff (28 Hz, slope 24 dB/Oct) and high cutoff (499 Hz, slope 24 dB/Oct, and Notch (50 Hz). After segmentation according to the six conditions (N_NoCue, N_Cue, P_NoCue, P_Cue, U_NoCue, U_Cue), we rectified and smoothed the data (moving average of 16 ms) and applied baseline correction (50 ms prior to onset of the startle tone). Startle amplitudes were defined as peak magnitudes between 20 and 150 ms after the probe onset. Following automatic exclusion of trials with strong baseline noise (> 5 μV before tone onset), the remaining trails were manually checked for artifacts. Amplitudes smaller than 2 μV were defined as nonresponses and set to zero (Blumenthal et al. 2005). Overall nonresponse was labelled in case of less than six trials evaluable per condition or less than 50% of evaluable startle responses in total (Lieberman et al. 2017). Before analysis, startle data were transformed into *T* scores ([*Z* scores × 10] + 50) to account for interindividual variability of general startle reactivity (Levenston et al. 2000; Grillon et al. 2013). Startle to P-threat was defined as startle during P_Cue adjusted for N_Cue, and startle to U-threat was defined as startle during U_Cue adjusted for N_Cue (Stevens et al. 2019). We decided to apply residualized change scores since they have better psychometric properties than difference scores (Meyer et al. 2017). Data of *n* = 4 for the etifoxine group, *n* = 4 for the alprazolam group, and *n* = 4 for the placebo group were not included to analysis because of technical issues with the experiment or identification as nonresponders (for more detailed information, see supplementary Fig. S1).

*Self-reports*: The anxiety ratings that were administered twice during the NPU-threat task were averaged across the two assessment time points. As for startle reactivity, we computed residualized change scores (Meyer et al. 2017) for further analysis, with P-threat being defined as stated anxiety during P_Cue adjusted for N_Cue and U-threat as stated anxiety during U_Cue adjusted for N_Cue.

*Attention measures*: During the CPT-AX, we assessed the mean reaction time for correct answers to the probe in AX trials (RT_AX_hits in ms). The values were exported separately for each of the four blocks using MATLAB (version R2017b, MathWorks, Natick, Massachusetts, USA). Data of *n* = 1 of the alprazolam group were excluded from further analysis because of issues with the experiment.

*TSPO gene polymorphism rs6971*: Using 4 ml whole blood samples of the screening day, we determined the presence of the TSPO gene polymorphism for subjects of the etifoxine group (Bahr et al. 2021). This was done with the QIAamp DNA Mini Kit (Qiagen, Hilden, Germany), optical absorbance, gel electrophoresis, polymerase chain reaction, and sequencing according to the Sanger method (Sanger et al. 1977) (for the whole procedure, see Bahr et al. 2021).

### Statistical analyses

Statistical analyses were conducted using SPSS (version 25, IBM Statistics). The significance level was set at *α* = 0.05. Univariate analyses of variance (ANOVA) were performed to test for group differences concerning the psychometric trait and demographic variables as well as the attention measures of the screening day.

Before computing any repeated measures ANOVA, we tested for homogeneity of variances using Levene’s tests, and in case of violation of sphericity, we report Greenhouse–Geisser (GG) corrected values. Significant main effects or interactions were followed up by Bonferroni-corrected post hoc *t*-tests or univariate ANOVAs.

To examine the effects of the NPU-threat task on startle reactivity and self-reports, we conducted repeated measures ANOVAs with *condition* (N, P, U) and *cue* (NoCue, Cue) as within-subjects factor separately for the two testing days.

To analyze the effects of the medication on P-threat responding, we computed repeated measures ANOVAs with the within-subjects factor *day* (day 1, day 5) and the between-subjects factor *treatment* (alprazolam, etifoxine, placebo) for the startle and the self-reported anxiety to P-threat, respectively. For the analysis of the effects of treatment on responses to U-threat, we computed repeated measures ANOVAs with the within-subjects factor *day* (day 1, day 5) and the between-subjects factor *treatment* (alprazolam, etifoxine, placebo) for the startle and the self-reported anxiety to U-threat, respectively. In addition to the *T*-transformed values, we repeated those analyses with the raw startle data (see supplementary results).

To examine the impact of possible sedation, we computed ANCOVAS including the startle responses and self-reports to U-threat and P-threat as factors with the between-subjects factor *treatment* and RT_AX_hits as covariate separately for days 1 and 5 of treatment.

Using moderation analyses (PROCESS macro; Hayes 2018), we checked whether trait anxiety (STAI trait, ASI-3, IUS-18) moderates the effects of treatment on startle responses to threat on the two testing days. For significant interactions, we applied the Johnson-Neyman technique to identify the range of values of the moderator where those interactions reached significance (Hayes and Montoya 2017).

To examine the impact of the TSPO polymorphism, we repeated analyses for the startle measurements related to P-threat and U-threat responding after exclusion of participants of the etifoxine group that were homozygous for the polymorphism.

## Results

### Study sample

A total of 82 interested subjects were screened for eligibility of whom 60 met inclusion criteria and were randomized to one of the three groups (for the CONSORT flowchart, see supplementary Fig. S1). Participants were aged between 18 and 48 years (*M* = 27.77, *SD* = 6.92). Baseline demographic and psychometric characteristics of the total sample did not differ significantly between the three groups (see Table 1).

**Table 1:** Statistics for physical variables of the study sample

Variable	Placebo	Alprazolam	Etifoxine	Statistics		
	*M* ± *SD*	*M* ± *SD*	*M* ± *SD*	*F* ratio	*df*	*p*
Age (years)	26.05 ± 4.71	27.55 ± 6.51	29.70 ± 8.78	1.43	2,57	0.249
Height (cm)	180.55 ± 5.67	179.30 ± 7.66	179.0 ± 5.80	0.27	2,57	0.762
Weight (kg)	77.50 ± 11.83	77.55 ± 16.17	77.41 ± 10.07	0.00	2,57	0.999
BMI (kg/m^2^)	23.75 ± 3.13	23.96 ± 3.51	24.07 ± 2.65	0.05	2,57	0.949

Data represent mean (*M*), standard deviation (*SD*), and results of the univariate ANOVAs for the three experimental groups. *BMI*, body mass index

### Startle response

**Fig. 1:**
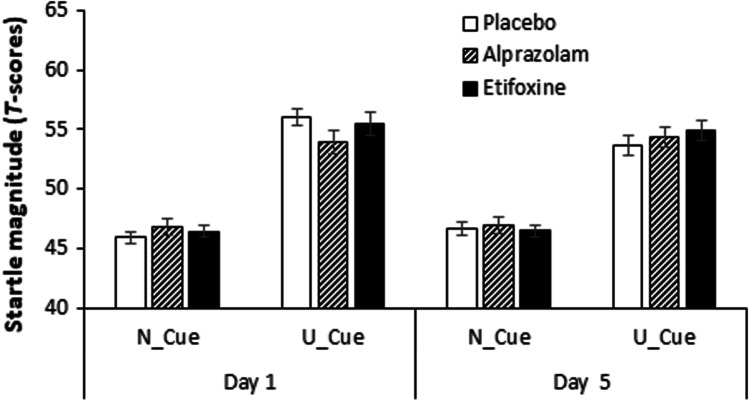
Startle response to U-threat in the NPU-threat test. Overview of startle responses to U-threat (*T* scores) separately for the three groups and the two treatment days. Startle potentiation to U-threat was defined as startle during the unpredictable condition when the cue was present (U_Cue) adjusted for startle during the neutral condition when the cue was present (N_Cue) (Stevens et al. 2019). Error bars show standard errors:

**Fig. 2:**
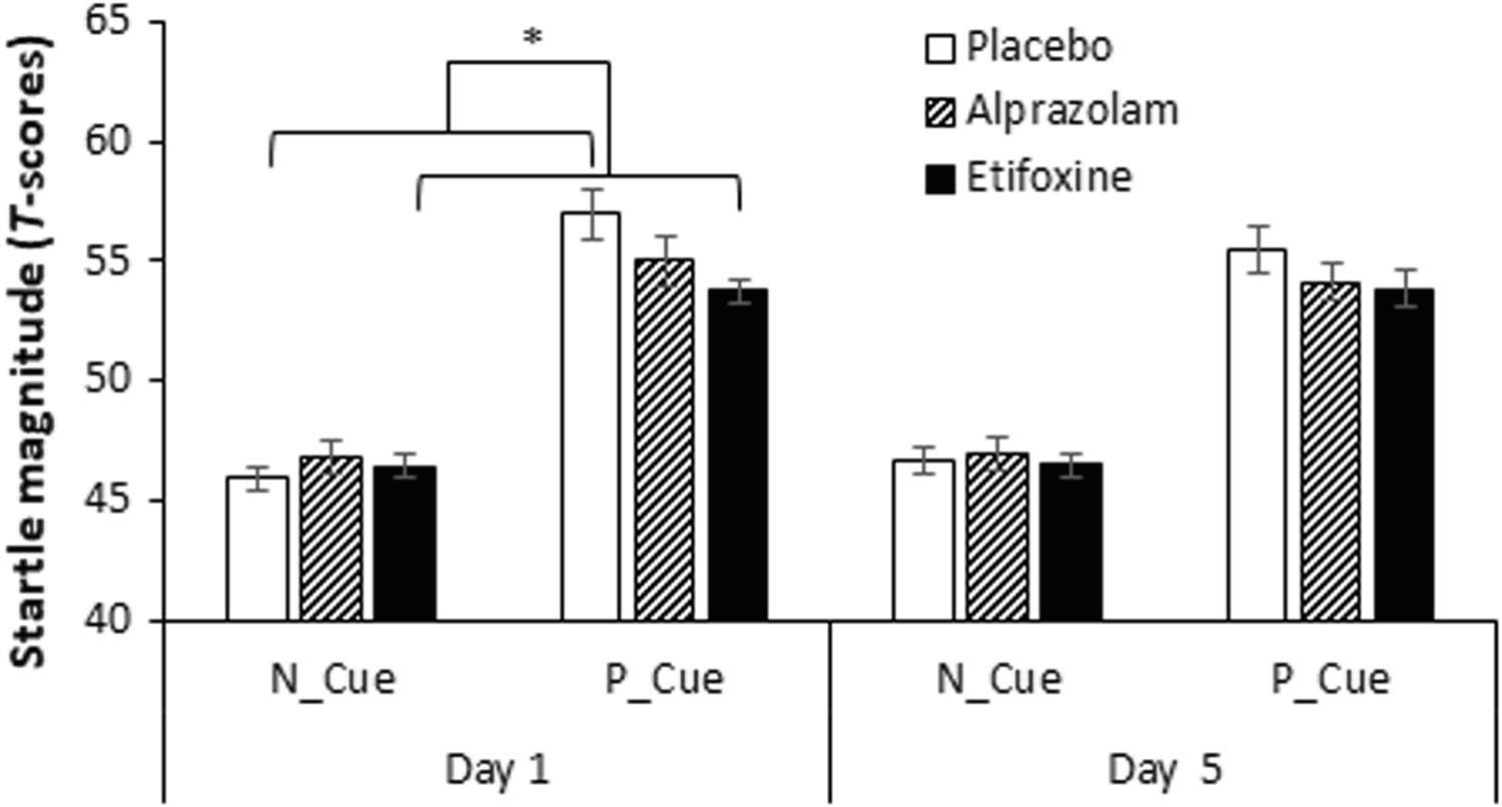
Startle response to P-threat in the NPU-threat test. Overview of startle responses to P-threat (*T* scores) separately for the three groups and the two treatment days. Startle potentiation to P-threat was defined as startle during the predictable condition when the cue was present (P_Cue) adjusted for startle during the neutral condition when the cue was present (N_Cue) (Stevens et al. 2019). Error bars show standard errors. *p < .05

Skewness and kurtosis values of the raw startle responses for each condition and each treatment are presented in the supplementary Table S2. The *T*-transformed startle responses differed between the three conditions on day 1 (*F*(2.94) = 98.47, *p* < 0.001, *ηp*^2^ = 0.68) and day 5 (*F*(2.94) = 98.87, *p* < 0.001, *ηp*^2^ = 0.68) (see Figs. 1 and 2). There was a main effect of *cue* on day 1 (*F*(1.47) = 98.97, *p* < 0.001, *ηp*^2^ = 0.68) and day 5 (*F*(1.47) = 89.7, *p* < 0.001, *ηp*^2^ = 0.66) as well as a significant interaction *condition* × *cue* interaction on day 1 (*F*(1.73, 81.26) = 49.0, *p* < 0.001, *ηp*^2^ = 0.51) and day 5 (*F*(1.54, 72.35) = 32.12, *p* < 0.001, ηp^*2*^ = 0.41).

Startle responses to U-threat did not change across the days (*day*, *F*(1.45) = 0.0, *p* = 1.00, *ηp*^2^ = 0.00) and not vary between groups (*treatment*, *F*(2.45) = 0.45, *p* = 0.642, *ηp*^2^ = 0.020). Furthermore, there was no significant interaction between *day* × *treatment* (*F*(2.45) = 1.21, *p* = 0.309, *ηp*^2^ = 0.051) (see Fig. 1).

Startles to P-threat did not differ between the two testing days (*day*, *F*(1.45 = 0.0), *p* = 1.0, *ηp*^2^ = 0.00. However, there was a significant effect of treatment on startle potentiation to P-threat (*treatment*, *F*(2.45) = 3.86, *p* = 0.028, *ηp*^2^ = 0.15). Follow-up analyses revealed a significant reduction of startle response by etifoxine (− 0.77, 95% CI [− 1.47, − 0.08], *p* = 0.024) but not by alprazolam (− 0.39, 95% CI [− 1.08, 0.31], *p* = 0.519) in comparison with placebo. Moreover, although the interaction *day* × *treatment* was not significant (*F*(2.45) = 0.22, *p* = 0.801, *ηp*^2^ = 0.01), an exploratory analysis revealed a significant reduction of startle responses to P-threat in the etifoxine group only for day 1 of treatment (− 0.86, 95% CI [− 1.69, − 0.03], *p* = 0.041) but not for day 5 of treatment (− 0.70, 95% CI [− 1.55, 0.15], *p* = 0.142) (see Fig. 2).

For the analyses of startle responsivity using raw data, there were no remarkable changes except for the treatment effect on P-threat, which was now found significant for alprazolam (− 0.73, 95% CI [− 1.39, − 0.07], *p* = 0.027) but not for etifoxine (− 0.47, 95% CI [− 1.13, 0.19], *p* = 0.251) in comparison with placebo (for all results, see supplementary material).

### Self-reports

**Fig. 3:**
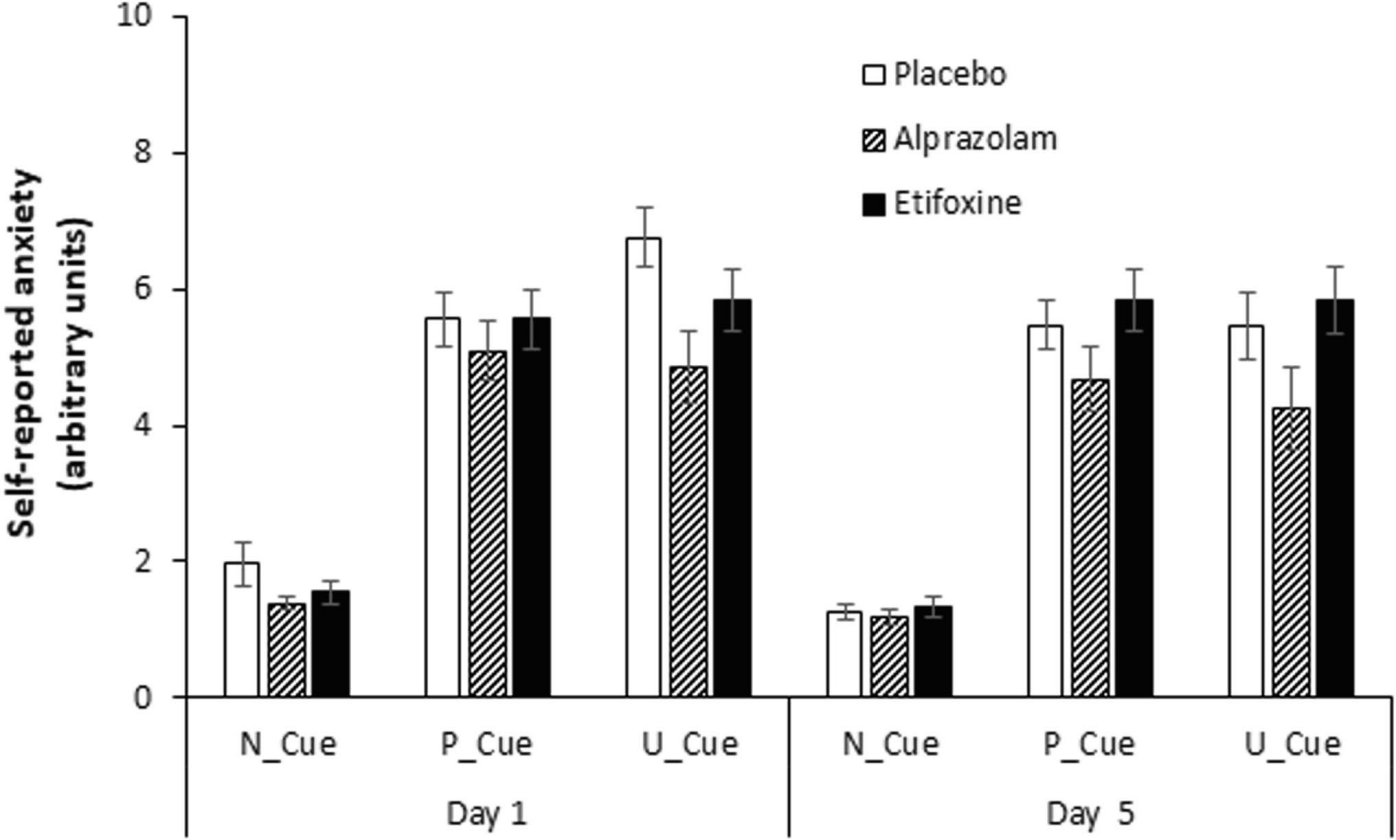
Self-reported anxiety during presentation of the cue for the three conditions of the NPU-threat test. Overview of self-reported anxiety (range from 0 to 10) to the three conditions (neutral, predictable, unpredictable) for the intervals when the cue was present (N_Cue, P_Cue, U_Cue) for the three groups and the two treatment days. Error bars show standard errors

Skewness and kurtosis values of the self-reports for each condition and each treatment are presented in the supplementary Table S3. For self-reports on anxiety, there was a main effect of *condition* on day 1 (*F*(1.61, 85.49) = 215.40, *p* < 0.001, *ηp*^2^ = 0.80) and day 5 (*F*(1.33, 70.71) = 214.96, *p* < 0.001, *ηp*^2^ = 0.80) (see Fig. 3) as well as a main effect of *cue* on day 1 (*F*(1.53) = 121.37, *p* < 0.001, *ηp*^2^ = 0.70) and day 5 (*F*(1.53) = 109.40, *p* < 0.001, *ηp*^2^ = 0.67). Those were qualified by a *condition* × *cue* interaction on day 1 (*F*(1.41, 74.52) = 112.28, *p* < 0.001, *ηp*^2^ = 0.68) and day 5 (*F*(1.41, 74.58) = 154.75, *p* < 0.001, *ηp2* = 0.75).

Self-reports on U-threat did not change across the days (*day*, *F*(1.45) = 0.0, *p* = 1.00, *ηp*^2^ = 0.00) and not vary between groups (*treatmen*t, *F*(2.45) = 0.0, *p* = 1.00, *ηp*^2^ = 0.00). Furthermore, there was no significant interaction between *day* × *treatment* (*F*(2.45) = 0.0, *p* = 1.00, *ηp*^2^ = 0.0) (see Fig. 3).

Self-reports on U-threat did not change across the days (*day*, *F*(1.45) = 0.0, *p* = 1.00, *ηp*^2^ = 0.00) and not vary between groups (*treatmen*t, *F*(2.45) = 0.0, *p* = 1.00, *ηp*^2^ = 0.00). Furthermore, there was no significant interaction between *day* × *treatment* (*F*(2.45) = 0.0, *p* = 1.00, *ηp*^2^ = 0.0) (see Fig. 3).

### Sedation

After adjusting for RT_AX_hits, the treatment effects on the startle responses did neither change for U-threat *F*(2.44) = 0.59, *p* = 0.556, *ηp*^2^ = 0.03 and P-threat *F*(2.44) = 3.29, *p* = 0.046, *ηp*^2^ = 0.13 on day 1 of treatment nor on day 5 with U-threat *F*(2.44) = 0.54, *p* = 0.542, *ηp*^2^ = 0.02 and P-threat *F*(2.44) = 2.07, *p* = 0.139, and *ηp*^2^ = 0.09.

### Impact of trait anxiety

For anxiety sensitivity (ASI-3), the overall model was significant for U-threat on day 1, *F*(3.44) = 3.79, *p* = 0.017, predicting 12.49% of the variance. The ASI-3 significantly moderated the effect between pharmacological treatment and U-threat startle day 1, *ΔR*^2^ = 10.96%, *F*(1.44) = 10.57, *p* < 0.01, 95% CI [0.017, 0.075]. The Johnson-Neyman technique yielded that this interaction was significant for ASI-3 scores below 8.67 (*t* =  − 2.02, *p* = 0.05) or above 23.76 (*t* = 2.02, *p* = 0.05) as was the case for in total 48% of the scores. For all results, see supplementary material.

### TSPO gene polymorphism rs6971

Out of the 20 subjects of the etifoxine group, *n* = 12 were identified as high-affinity binders (GG), while *n* = 5 were mixed-affinity binders (AG), and *n* = 3 were low-affinity binders (AA) (Bahr et al. 2021). Neither for responses to U-threat nor to P-threat main effects or interactions were altered after exclusion of the three participants that were homozygous for the polymorphism rs6971 (see supplementary Table S4).

## Discussion

Within the present work, we experimentally compared the acute and short-term effects of the TSPO ligand etifoxine and the benzodiazepine alprazolam on predictable (P-threat) and unpredictable threat (U-threat) responding in healthy males using a standardized startle-based paradigm. None of the anxiolytic substances attenuated responses to U-threat on any of the testing days. Interestingly, the startle response to P-threat was reduced by etifoxine on the first treatment day. However, deviant results were shown for raw data analysis yielding a significant effect only for alprazolam. Overall, there were no hints of anxiolytic effects in 17 or an impact of sedation by either medication.

Tentatively, the effect on P-threat startle in the transformed data might hint on a potential indication of etifoxine for the treatment of specific subtypes of anxiety disorders even if our results relay on a healthy sample. While previous startle-based research has associated most of the common subtypes to U-threat (Gorka et al. 2017), recent research reported elevated responses to P-threat in social anxiety disorder (Grillon et al. 2017). For etifoxine, preclinical research reported therapeutic effects after infusion to the basolateral amygdala (Zeitler et al. 2016), a region that strongly interacts with the central nucleus of the amygdala, the major player in specific fear (Davis & Whalen, 2001; Walker et al. 2003). Further hints linking TSPO to specific fear come from research in humans that showed reduced pharmacologically induced panic after 7 days intake of the selective TSPO ligand XBD-173 in comparison with placebo (Rupprecht et al. 2009). In patients, efficacy of etifoxine at a level comparable to that of benzodiazepines has been reported for adjustment disorders with anxiety (Nguyen et al. 2006; Servant et al. 1998; Stein, 2015, 2018), which implies the appearance of clinical symptoms within a period of 6 months after the experience of specific stressful events (Vanin & Helsley, 2008). Our results on etifoxine for startle potentiation to P-threat together with (pre-) clinical findings of effects on particular components of anxiety call for further studies that investigate not only a possible specific indication of the ligand but also the use of TSPO as a biological disease marker.

Nevertheless, our findings are preliminary and should be extended in future studies, especially since the effects of etifoxine on P-threat startle did not endure the repetition of analyses with raw data, the use of which has been set out as preferable by some work (Bradford et al. 2015). Instead, those analyses yielded an effect on P-threat startle on day 1 for alprazolam — a finding that is not in line with prior research (Grillon et al. 2006) and thus has to be confirmed in future work. Anyway, due to the design, the missing measurement of baseline, and general startle responsivity as well as extreme skewness/kurtosis values across all conditions especially for the alprazolam group, we put more emphasis on transformed data in the present work.

Concerning startle potentiation to U-threat, our findings stand in contrast to some of the previous findings on GABAergic substances (Grillon et al. 2006; Riba et al. 2001; Graham et al. 2005; Scaife et al. 2005), which, however, have already been challenged before (Acheson et al. 2012; Baas et al. 2009). Etifoxine and alprazolam showed comparable anxiolytic effects in patient studies at exactly the dosages that we chose for the present work (Stein, 2015). Nevertheless, they might have been too small with respect to modulation of startle reactivity in healthy subjects. While 0.5 mg alprazolam have already been shown effective for the reduction of startle responses to P-threat (Riba et al. 2001), most of the studies applied at least 1 mg, and also, dose-dependent effects have been revealed (Grillon et al. 2006). Although refuted by some work (Grillon et al. 2006), it cannot be ruled out that former findings on higher dosages have at least partly been due to muscle relaxing or sedating effects, which turned out negligible in our study. Likewise, for etifoxine, dose dependency of effects on physiological responses to an anxiety-related context has been shown in preclinical research (Verleye.

Furthermore, it is possible that effects of the applied dosages only occur in more anxious subjects. Anxiety ratings on P-threat and U-threat, which were stated on a scale from 1 to 10, did, on average, not exceed values of 7 in any of the groups. Regarding trait anxiety measures, our subjects ranged in the lower/medium range and thus are comparable to previous work (Grillon et al. 2006). Out of the assessed trait anxiety measures, only anxiety sensitivity was shown to moderate the effects of treatment on U-threat startle on day 1 for scores in certain ranges. This trait has been linked to startle reactivity (Nelson et al. 2015) as well as increased risk for the development of anxiety disorders (Allan et al. 2014) before and should therefore be kept in mind for future studies, e.g., by a priori classification according to low and high scores. In general, our investigations should be transferred to samples of patients suffering from anxiety and other stress-related disorders, as those show deviant reactivity, most of all, elevated startle responses to U-threat (Grillon et al. 2008; Pole et al. 2003). Furthermore, it must be noted that only males were included in the present study, while previous work was based on gender mixed samples with an even higher rate of females in some studies (Acheson et al. 2012; Grillon et al. 2006; Riba et al. 2001) which might at least partly explain the deviant results.

The fact that self-reported anxiety related to U-threat and P-threat were not affected by any of the anxiolytic treatment is in line with previous studies that missed attenuating effects of anxiolytic or antidepressant compounds on respective self-reports (Grillon et al. 2006; Grillon et al. 2007). Pharmacological compounds exert effects on a molecular level that are often reflected in changes of physiological parameters without being expressed in subjective measurements in healthy subjects. Since verbal reports of emotions involve a certain amount of cognitive activity, they might just be unable to uncover early drug effects. Since the subjective ratings were acquired retrospectively, subtle differences in responding to the six different conditions may have been concealed by the time elapsed. Therefore, further research should consider online assessment of the subjective state.

Regarding the TSPO gene polymorphism rs6971, our sample represent the overall distribution of the so called ow-affinity binders with around 10% among the Europeans population (Kreisl et al. 2013). Not revealing an impact of this genetic variation on startle responsivity might be due to the small number of subjects and a resulting statistical type II error. Follow-up research should further address the relevance of structural changes of TSPO for the effects of etifoxine, e.g., by prior stratification to the presence of the genetic variant.

Although all methodological requirements were set to adhere to prior work on pharmacological startle-related research as largely as possible, one might critically question the between-subjects design of the present work (Grillon et al. 2006; Riba et al. 2001). Thus, possible group differences on general startle reactivity prior to administration of the substances might have biased our results, and baseline measurement as part of the screening might be essential to exclude subjects who do not display threat-potentiated startle. Indeed, the present work did include comparisons within subjects, as we administered the task for a second time after 5 days of treatment. In line with previous research, the paradigm has proven to be suitable for repeated measures, as startle reactivity during the unpredictable condition still exaggerated that of the neutral (safe) context at the second session (Klumpers et al. 2010). Although the cue was predictive only in the P condition, the fact that subjects were not shocked at every presentation of the cue during that condition might limit its predictability. Follow-up studies might profit from modifications like reliable appearance of the shock at the end of a countdown for P-threat (Gorka et al. 2017) or the use of alternating threat probabilities (Bradford et al. 2014) with a shock reinforcement rate of 75% eliciting greater startle responses than a rate of 50%, which we used in the present work (Chin et al. 2016).

In conclusion, using a startle-based paradigm, we revealed different effects of the TSPO ligand etifoxine and the benzodiazepine alprazolam on startle potentiation to P-threat and U-threat in healthy subjects. While none of the substances had an impact on startle responses to U-threat on any of the two testing days, etifoxine attenuated the startle response to P-threat on the first day of treatment, although contrary results were found with raw data analysis. A possible indication of etifoxine for the acute treatment of specific subtypes of anxiety disorders should be followed-up in upcoming research. Future studies might apply versions of the paradigm that are compatible with neuroimaging to forward understanding of underlying mechanisms or imply dose-dependent comparisons and online assessment of subjective experience to further unravel the particular contribution of sedation and anxiolysis.

## Supplementary Information

Below is the link to the electronic supplementary material.

Supplementary file1 (RTF 3239 KB)
