# Supplementary material for: Differential effects of the translocator protein 18 kDa (TSPO) ligand etifoxine and the benzodiazepine alprazolam on startle response to predictable threat in a NPU-threat task after acute and short-term treatment
Source: Psychopharmacology (Berl). 2022 Mar 12;239(7):2233–44. doi: 10.1007/s00213-022-06111-x (PMC9205810; doi:10.1007/s00213-022-06111-x)
Supplement: Supplementary file 1 — Supplementary file1 (RTF 3239 KB) [file 213_2022_6111_MOESM1_ESM.rtf]

Supplementary Material

Figure S1. CONSORT flowchart of participants

Flow diagram of the progress through the phases of the parallel randomized trial including three groups (placebo, alprazolam, etifoxine) depicting enrollment, randomized intervention allocation, follow-up, and data analysis.


Table S2. Skewness and kurtosis of the raw startle responses for the three conditions on day 1 and 5
	Neutral	Predictable	Unpredictable	
	NoCue	Cue	NoCue	Cue	NoCue	Cue	
	Ske	Kur	Ske	Kur	Ske	Kur	Ske	Kur	Ske	Kur	Ske	Kur	
PLC_D1	1.21	1.02	1.83 	3.78	0.99	-0.16	1.30 	0.47	0.97	0.30	1.21	0.14	
PLC_D5	1.18	0.74	1.68	2.52	1.12	0.44	1.44	1.32	1.23	0.99	2.37	6.31	
ALP_D1	3.60	13.77	3.35	12.35	2.98	10.38	2.46	7.86	2.87	9.56	2.84	9.60	
ALP_D5	2.84	9.68	3.35	12.39	3.16	11.46	1.90	5.71	1.31	2.70	2.14	6.07	
ETX_D1	0.77	-0.77	0.79	-0.50	1.11	0.11	0.72	-0.67	0.37	-1.26	0.54	-1.14	
ETX_D5	1.0	-0.23	1.19	0.42	0.92	-0.49	0.81	-0.69	0.95	0.13	0.71	-1.03	

Overview of skewness (Ske) and kurtosis (Kur) of the raw startle responses for the three conditions (neutral, predictable, unpredictable) when the cue was shown (Cue) or absent (NoCue) for the three experimental groups on day 1 (D1) and day 5 (D5) of treatment. PLC = placebo, ALP = alprazolam, ETX = etifoxine.


Table S3. Skewness and kurtosis of the anxiety ratings for the three conditions on day 1 and 5
	Neutral	Predictable	Unpredictable	
	NoCue	Cue	NoCue	Cue	NoCue	Cue	
	Ske	Kur	Ske	Kur	Ske	Kur	Ske	Kur	Ske	Kur	Ske	Kur	
PLC_D1	2.13	4.18	1.97	4.13	0.50	-1.06	0.05	0.39	-0.08	-0.56	-0.43	-0.29	
PLC_D5	1.59	1.69	1.76	2.16	0.98	0.69	0.03	-0.12	-0.20	-1.62	0.55	.10	
ALP_D1	0.76	-0.86	0.50	-1.36	0.98	0.13	-0.02	-0.62	0.50	-0.75	0.44	-1.30	
ALP_D5	3.56	13.05	2.51	5.73	1.45	0.53	0.08	-0.55	0.06	-0.16	0.48	-0.62	
ETX_D1	1.73	2.76	1.97	4.49	-0.39	-1.25	-0.61	0.03	-1.10	1.58	-0.69	.65	
ETX_D5	2.49	5.27	1.81	1.77	0.91	-0.27	-0.92	0.32	-1.05	1.35	-0.91	0.06	

Overview of skewness (Ske) and kurtosis (Kur) of the anxiety ratings for the three conditions (neutral, predictable, unpredictable) when the cue was shown (Cue) or absent (NoCue) for the three experimental groups on day 1 (D1) and day 5 (D5) of treatment. PLC = placebo, ALP = alprazolam, ETX = etifoxine.


Table S4. Results of analysis of startle data after exclusion of the TSPO gene polymorphism rs6971
Parameter	Source		F ratio		df		p		np2	
P-threat	Day		0.001		1,43		.975		.00	
	Treatment		2.88		2,43		.067		.12	
	Day x treatment		0.23		2,43		.800		.01	
U-threat	Day		0.01		1,43		.917		.00	
	Treatment		0.23		2,43		.792		.01	
	Day x treatment		1.18		2,43		.316		.05	

Results of the repeated measures ANOVAs for startle response related to P-threat and U-threat after exclusion of the subjects of the etifoxine group that were homozygous for the TSPO gene polymorphism rs6971 (n = 3). 


Supplementary results
Raw data analysis
Analyses on raw startle data to U-threat yielded no changes of responses across the days (day, F(1,45) = 0.04, p = .846, çp2  = .001) or between groups (treatment, F(2,45) = 1.91, p = .160, çp2  = .078). Further, there was no significant interaction between day x treatment (F(2,45) = 2.47, p = .096, çp2  = .099).
Raw values of startle responses to P-threat did not differ between the two testing days (day, F(1,45 = 0.17), p = .682, çp2  = .004. As for the transformed data, there was a significant effect of treatment on startle potentiation to P-threat (treatment, F(2,45) = 3.85, p = .029, çp2 = .15). Follow-up analyses, however, revealed a significant reduction of startle response by alprazolam (-.73, 95%-CI[-1.39, -.07], p = .027) but not by etifoxine (-.47, 95%-CI[-1.13, .19], p = .251) in comparison to placebo. Although the interaction day x treatment was not significant (F(2,45) = 0.99, p = .379, çp2 = .04), a follow-up analysis showed a significant reduction of startle responses to P-threat in the alprazolam group only for day 1 of treatment (-.87, 95%-CI[-1.66, -.07], p = .028) but not for day 5 of treatment (-.58, 95%-CI[-1.23, .07], p = .092). 

Moderation analysis for trait anxiety
For trait anxiety (STAI-trait) the overall model of the moderation analysis was significant only for P-threat on day 1 of treatment, F(3,43) = 3.01, p = .040, predicting 15.76% of the variance. The STAI trait did not significantly moderate the effect between pharmacological treatment and P-threat startle day 1, ÄR² = 3.62%, F(1,43) = 0.55, p = .179, 95% CI[-0.084, 0.015]. The overall model of the moderation analysis for the STAI-trait was neither significant for U-threat startle day 1, R² = 0.24%, F(3,43) = 0.04, p = .987, nor for U-threat startle day 5, R² = 3.77%, F(3,43) = 0.55, p = .651, or P-threat startle day 5, R² = 10.67%, F(3,43) = 1.86, p = .151. Trait anxiety did not significantly moderate the effect between pharmacological treatment and U-threat day 1, ÄR² = 0.21%, F(1,43) = 0.08, p = .785, 95% CI[-0.048, 0.074], U-threat day 5, ÄR² = 0%, F(1,43) = 0.0, p = .993, 95% CI[-0.062, 0.066], or P-threat day 5, ÄR² = 0.92%, F(1,43) = 0.31, p = .581, 95% CI[-0.079, 0.046]. 
For intolerance of uncertainty (IUS-18) the overall model was significant only for P-threat on day 1 of treatment, F(3,43) = 3.73, p = .018, predicting 20% of the variance. The IUS-18 did not significantly moderate the effect between pharmacological treatment and P-threat startle day 1, ÄR² = 20%, F(1,43) = 1.07, p = .306, 95% CI[-0.033, 0.015]. The overall model of the moderation analysis for the IUS-18 was neither significant for U-threat startle day 1, R² = 0.64%, F(3,43) = 0.25, p = .860, nor for U-threat startle day 5, R² = 10.09%, F(3,43) = 0.79, p = .505, or P-threat startle day 5, R² = 10.45%, F(3,43) = 1.72, p = .177. Intolerance of uncertainty did not significantly moderate the effect between pharmacological treatment and U-threat day 1, ÄR² = 0.56%, F(1,43) = 0.39, p = .537, 95% CI[-0.020, 0.033], U-threat startle day 5, ÄR² = 7.57%, F(1,43) = 1.65, p = .206, 95% CI[-0.068, 0.009], or P-threat startle day 5, ÄR² = 1.32%, F(1,43) = 0.31, p = .579, 95% CI[-0.048, 0.035]. 
For anxiety sensitivity (ASI-3) the overall model of the moderation analysis was neither significant for U-threat startle day 5, R² = 4.87%, F(3,44) = 0.91, p = .442, nor for P-threat startle day 1, R² = 12.95%, F(3,44) = 1.91, p = .142, or P-threat startle day 5, R² = 10.3%, F(3,44) = 1.57, p = .211. Anxiety sensitivity did not significantly moderate the effect between pharmacological treatment and U-threat day 5, ÄR² = 0%, F(1,44) = 0.006, p = .938, 95% CI[-0.047, 0.037], P-threat day 1, ÄR² = 0%, F(1,44) = 0.03, p = .866, 95% CI[-0.044, 0.049], or P-threat day 5, ÄR² = 1.25%, F(1,44) = 0.32, p = .576, 95% CI[-0.063, 0.034].
